# Genome-Wide Characterization of *OFP* Family Genes in Wheat (*Triticum aestivum* L.) Reveals That *TaOPF29a-A* Promotes Drought Tolerance

**DOI:** 10.1155/2020/9708324

**Published:** 2020-11-07

**Authors:** Dezhou Wang, Zhichen Cao, Weiwei Wang, Wengen Zhu, Xiaocong Hao, Zhaofeng Fang, Shan Liu, Xiaoyan Wang, Changping Zhao, Yimiao Tang

**Affiliations:** ^1^Beijing Engineering and Technique Research Center for Hybrid Wheat, Beijing Academy of Agriculture and Forestry Sciences, Beijing 100097, China; ^2^College of Agriculture, Yangtze University, Jingzhou 434023, China

## Abstract

OVATE family proteins (OFPs) are plant-specific transcription factors that play important roles in plant development. Although common wheat (*Triticum aestivum* L.) is a major staple food worldwide, OFPs have not been systematically analyzed in this important crop. Here, we performed a genome-wide survey of *OFP* genes in wheat and identified 100 genes belonging to 34 homoeologous groups. *Arabidopsis thaliana*, rice (*Oryza sativa*), and wheat *OFP* genes were divided into four subgroups based on their phylogenetic relationships. Structural analysis indicated that only four *TaOFPs* contain introns. We mapped the *TaOFP* genes onto the wheat chromosomes and determined that *TaOFP17* was duplicated in this crop. A survey of cis-acting elements along the promoter regions of *TaOFP* genes suggested that subfunctionalization of homoeologous genes might have occurred during evolution. The *TaOFPs* were highly expressed in wheat, with tissue- or organ-specific expression patterns. In addition, these genes were induced by various hormone and stress treatments. For instance, *TaOPF29a-A* was highly expressed in roots in response to drought stress. Wheat plants overexpressing *TaOPF29a-A* had longer roots and higher dry weights than nontransgenic plants under drought conditions, suggesting that this gene improves drought tolerance. Our findings provide a starting point for further functional analysis of this important transcription factor family and highlight the potential of using *TaOPF29a-A* to genetically engineer drought-tolerant crops.

## 1. Introduction

The shape of produce is an important agronomic trait. Identifying and characterizing the genes that regulate this trait in crops could lead to the improvement of this agriculturally important trait. The first OVATE gene cloned from plants controls fruit shape in tomato (*Solanum lycopersicum*) [1]. OVATE family proteins (OFPs) have recently been identified in a variety of land plants [2]. OFPs are plant-specific transcriptional regulators containing a common domain designated as the OVATE domain or Domain of Unknown function 623 (DUF623) [3, 4]. These proteins are widely involved in the growth and development of multiple plant tissues and organs, contributing to processes such as secondary cell wall formation, cell elongation, cotyledon development, floral shape formation, and brassinolide (BR) signal transduction [5]. Moreover, a growing body of evidence suggests that OFPs participate in abiotic stress pathways [2, 6, 7].

Many OFP genes have been identified in diverse plant species, including 31 in rice (*Oryza sativa*) [8], 28 in apple (*Malus domestica*) [9], 18 in *Arabidopsis thaliana* [10], 19 CsOFPs in cucumber (*Cucumis sativus*) [11], 18 CmOFPs in melon [12], 17 ClOFPs in watermelon [11], and 35 RsOFPs in radish [2], laying the foundation for identifying new OFP genes in other plant species. Subsequent studies in Arabidopsis have indicated that OFPs affect various aspects of plant growth and development, likely by interacting with different types of transcription factors and functional proteins. AtOFP1 functions in the gibberellin (GA) signaling pathway by regulating *AtGA20ox1* expression [4]. AtOFP1 and AtOFP4 interact with KNAT7 to regulate secondary cell wall formation in Arabidopsis [13]. AtOFP5 negatively regulates the activity of the BLH1-KNAT3 complex, thereby affecting early embryo sac development [14]. Loss-of-function alleles of *AtOFPs* did not display any morphological defects, but plants overexpressing *AtOFP* genes had smaller hypocotyls, siliques, leaves, rosettes, and floral organs than the wild type (WT) [4]. Tomato plants overexpressing *SlOFP20* showed several phenotypic defects, including altered fruit shape and floral architecture and reduced male fertility [15]. Among other horticultural crops, *RsOFP2.3* is related to tuberous root shape formation in radish, *StOFP20* controls tuber shape in potato (*Solanum tuberosum*), *CmOFP1a* is a candidate for the fruit size/fruit shape QTL *CmFS8.3*/*CmFSI8.3* in melon [12], and *CsOFP1a* and *ClOFP1a* are top candidates for two QTLs related to fruit shape in cucumber and watermelon [11]. *OFPs* play multiple roles in plants, including development, phytohormone signaling, and responses to stresses such as cold, salinity, and drought stress. In rice, OsOFP1 regulates the levels of proteins involved in BR responses and thereby modulates plant architecture and grain morphology. Overexpressing *OsOFP2* in rice resulted in reduced plant height, altered leaf and seed morphology, and changes in the positions of stem vascular bundles. Transcriptome analysis indicated that the expression of genes related to phylogeny, organogenesis, and hormone homeostasis was altered in these plants [16]. *OsOFP6* regulates plant development and confers resistance to drought and cold stress in rice. *OsOFP6* is involved in lateral root growth and initiation mediated by the auxin IAA. *OsOFP6* also functions in the determination of plant height and grain size, lateral root development, and abiotic stress responses [7]. *OsOFP8* regulates leaf angle via the BR signaling pathway, and *OsOFP30* is also involved in BR responses [17]. Overexpressing *OsOFP8* improved disease resistance, drought tolerance, and cold stress tolerance in rice [17]. *MdOFP04* and *MdOFP20* were significantly upregulated following NaCl treatment in apple (*Malus domestica*) [9].

Hexaploid bread wheat (*Triticum aestivum* L.), the most widely cultivated crop worldwide, contains three homologous subgenomes (A, B, and D) [18]. Although the wheat genome was recently sequenced and assembled, the *OFP* gene family has not been described in wheat. In the current study, we identified 100 *OFP* genes in wheat and comprehensively investigated these genes. We categorized the Arabidopsis, rice, and wheat *OFP* genes into four subclasses based on their phylogenetic relationships. We also analyzed the conserved motifs, structures, and duplication patterns of *OFP* genes in wheat and examined the syntenic relationships between wheat and rice *OFP* genes. Expression profiling in different tissues and in response to different stress treatments uncovered the possible roles of these genes in regulating plant development and responses to biotic and abiotic stress. We also performed a more detailed examination of the Ovate-like gene *TaOPF29a-A* (TraesCS1A02G387000). *TaOPF29a-A* is highly expressed in roots in response to drought stress and is likely involved in drought stress tolerance, as overexpressing this gene in wheat improved tolerance to drought treatment, highlighting its potential as a target for breeding stress-tolerant crops. The results of this study provide a reference for further functional analysis of *OFP*-related genes in crops.

## 2. Materials and Methods

### 2.1. Identification of Wheat *OFP* Gene Family Members

The wheat genome sequence was downloaded from Ensembl Plants (http://plants.ensembl.org/index.html). The Hidden Markov Model (HMM) profile of the OFP domain (PF04844) was downloaded from the Pfam website (http://pfam.xfam.org/) [19]. The OFP proteins were verified individually against the NCBI-CDD database (https://www.ncbi.nlm.nih.gov/Structure/cdd/wrpsb.cgi) [20], which was used to verify conserved OFP protein domains, remove OVATE domain-free proteins, and manually delete sequences without complete reading frames.

All protein sequences containing the OFP conserved domain were searched. To avoid missing OFP family members, we constructed a new OFP data set for wheat using a high-quality protein set (*E* value < 1 × 10^−20^) for multiple sequence alignment with Clustal (Clustal 2.1; https://www.ebi.ac.uk). Based on the aligned sequences, we constructed a new HMM with HMMER (HMMER 3.1; http://hmmer.org/) and used it as a query (*E* value < 0.01) to search against the *Triticum aestivum* genome sequencing data. Genes encoding proteins with OFP domains were identified as *OFP* gene candidates. The ExPASy online program (http://web.expasy.org/translate/) was used to analyze the physicochemical properties of the TaOFP proteins. The WoLF PSORT online program (https://wolfpsort.hgc.jp/) was used to predict the subcellular localizations of these proteins [21].

### 2.2. Sequence Analysis and Structural Characterization of TaOFPs

Bioinformatic analysis of *TaOFP* gene sequences and calculation of the coding sequence (CDS) length, molecular weight (MW), isoelectric point (pI), and open reading frame (ORF) length of each TaOFP were performed using the Compute pI/MW tool at the Expert Protein Analysis System (ExPASy) site (http://au.expasy.org/tools/pi-tool.html). Gene structures and conserved motifs were analyzed using GSDS (Gene Structure Display Server) and MEME (v.12.0; http://meme-suite.org/tools/meme), respectively. TBtools (TBtools-v0.53.jar) was used to analyze and visualize the structures (exons/introns) and conserved motifs of the *OFP* genes.2.3. Phylogenetic analysis and classification of the *TaOFP* gene family.

MEGA 7.0 was used to construct an individual phylogenetic tree of the *TaOFP* gene family [22]. The *AtOFP*, *OsOFP*, and *TaOFP* genes were divided into subfamilies based on their clustering patterns, and a comprehensive phylogenetic tree was constructed based on these genes using MEGA 7.0. All sequences were initially aligned using ClustalW (http://www.ebi.ac.uk/clustalw/) with default parameters [22]. Both phylogenetic trees were generated as described by Wang et al. [2] with MEGA 7.0 using the Neighbor-Joining method [23] with 1,000 bootstrap replicates [24].

### 2.3. Chromosomal Location Analysis and Duplication of *TaOFP* Genes

Each *TaOFP* gene was mapped onto the 21 wheat chromosomes in the IWGSC RefSeq v1.0 (cv. Chinese Spring) reference genome using Triticeae Multi-omics Center (http://202.194.139.32/). Ensembl (http://plants.ensembl.org/index.html) was used to extract information about chromosome length, and a physical map was drawn with MG2C (v2.1; http://mg2c.iask.in/mg2c-v2.1/). Segmental duplications and tandem duplications in the three subgenomes were separately identified using Clustal Omega (https://www.ebi.ac.uk/Tools/msa/clustalo/) [25]. The following criteria were used to identify the tandem duplication events of the *TaOFPs*: (1) alignment length was >80% of the complete gene sequence, (2) the aligned region had an identity of >80%, and (3) no genes were inserted between these genes. Segmental duplication was defined as follows: (1) alignment length was >1 kb and (2) the aligned region had an identity of >90% [26–29]. Ka/Ks ratios were calculated using Ka/Ks Calculator 2.0 (https://sourceforge.net/projects/kakscalculator2/) [30].

### 2.4. Analysis of Cis-Acting Elements in the *TaOFP* Gene Promoters

The cis-acting elements in the *TaOFP* promoters were analyzed using the TBtools software (v0.6669; http://cj-chen.github.io/tbtools/), which retrieved the upstream region (2.0 kb) of the CDS of each *TaOFP* from the wheat genome sequence and converted it into FASTA file format. The sequence was then submitted to PlantCARE (http://bioinformatics.psb.ugent.be/webtools/plantcare) [31] in batches. The results were filtered to retain the response-related cis-acting elements, including drought-responsive, auxin-responsive, jasmonate-responsive, abscisic acid-responsive, and GA-responsive elements. All related cis-elements were visualized using TBtools.

### 2.5. Expression Analysis of OFP Family Genes in Wheat

To further analyze the expression patterns of the OFP family genes in wheat, the CDS of each gene was submitted to the Wheat Expression Browser website to obtain expression data for different tissues of Chinese Spring wheat, and the expression patterns of OFP family members in wheat were systematically analyzed [32]. The *OFP* gene expression data were obtained from transcriptome data for Chinese Spring wheat seedlings under drought and heat stress conditions [33]. Chinese Spring wheat transcriptome data under GA, jasmonic acid, abscisic acid, salicylic acid, and cytokinin treatment were used to obtain *OFP* gene expression data from the Introduction to the Triticeae Multi-omics Center database [34]. The expression data were used to construct a heat map using TBtool [35].

### 2.6. Plant Transformation

The wheat (*T. aestivum*) cultivar Jingdong18 was used as the WT in this study. The full-length cDNA of *TaOFP29a-A* was cloned into pCUB to generate pCUB-TaOFP29a*-*A, which was recombined with the Ubi-TaOFP29a*-*A-3×FLAG-GFP vector to generate the overexpression construct. The pUbi::TaOFP29a*-*A vector was transformed into Jingdong18 via Agrobacterium-mediated transformation as described previously [36]. Wheat seeds were germinated in plates containing water. Plants were grown in a greenhouse at a constant temperature (20–25°C) under long-day conditions (16 h/8 h light/dark cycle). The sequences of the primer pairs used for vector construction are listed in Supplementary File 1.

### 2.7. Drought Stress Assay

Transgenic and wild type (WT) plants (*N* = 30) were grown for 4 weeks and then deprived of water for 2 weeks. To investigate drought tolerance, the plants were rewatered for one week, phenotypic changes were photographed, and the survival rate was calculated. There were three experimental replicates. In addition, three-day-old wheat seedlings were treated with 10% polyethylene glycol (PEG) 8000, and the expression levels of the *TaOFPs* were detected in whole plants at different time points [37].

### 2.8. RNA Extraction and qPCR Analysis

Total RNA was isolated from the samples using TRIzol reagent (Invitrogen). For qPCR, first-strand cDNA was synthesized using a Primer Script RT reagent Kit with gDNA Eraser (TaKaRa). The qPCR was conducted in a CFX™ real-time PCR detection system (Bio-Rad). The expression level of the wheat *Tubulin* gene was used as an endogenous control to normalize gene expression levels. Three biological replicates were performed for each treatment, and three technical replicates were performed for each biological replicate. Relative gene expression levels were calculated using the 2^–*ΔΔ*CT^ method [38]. The sequences of the primer pairs used for qPCR are listed in Supplementary File 1.

## 3. Results

### 3.1. Identification of OFP Family Genes in Wheat

We retrieved OFP transcription factor sequences from the wheat genome based on the HMM profile (PF04844) of the OFP family. Initially, 100 nonredundant putative *OFP* genes were identified. After removing the redundant forms of the genes, 100 genes were identified by HMM analysis (Supplementary File 2). The *TaOFP* genes were named sequentially from *TaOFP1-A* to *TaOFP31-D* based on homologous relationships between the wheat and rice genes. We analyzed the physicochemical properties of the *TaOFP* genes using the ExPASy online program. Detailed information about the OFP family genes in wheat, including the name and identifier (ID), number of amino acids in the encoded protein, pI, molecular weight (MW), ORF size, chromosome position, rice orthologs, and subcellular localization is provided in Supplementary File 3.

The amino acid composition and physicochemical properties differ among OFP family proteins, and the number of amino acids per protein varies greatly among subfamilies. The predicted sequences of the proteins encoded by the *TaOFP* genes ranged from 108 (*TaOFP24-D.2*) to 400 (*TaOFP30b-U*) amino acids in length, with an average length of 276 amino acids. The predicted MW ranged from 11,662 Da to 41,867.5 Da, with an average MW of 29,558.425 Da. The pI ranged from 4.08 (*TaOFP30b-B*) to 12.12 (*TaOFP23-D*), with an average of 8.8. The subcellular localizations of all the wheat OFPs could be predicted using the online software WoLF PSORT. Among these proteins, 43 were localized to the nucleus, 38 to the chloroplast, 7 to the cytosol, 6 to mitochondria, and 6 to the extracellular space, implying that wheat OFPs are involved in multiple biological processes.

### 3.2. Phylogenetic Analysis and Classification of the *OFP* Gene Family

**Figure 1:**
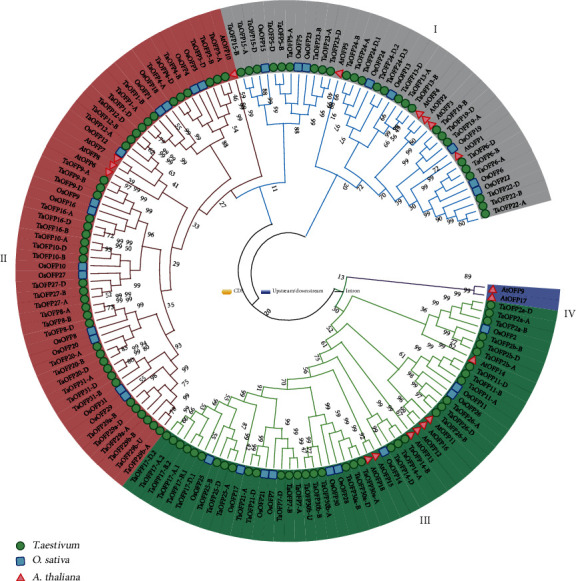
Phylogenetic tree of *OFP* genes from diverse species. The abbreviated species names are as follows: Ta, *Triticum aestivum*; At, *Arabidopsis thaliana*; and Os, *Oryza sativa*. The unrooted tree was generated using the MEGA 7.0 software. The bootstrap values are indicated on the branches. Sequence information is provided in Supplementary File 5.

**Figure 2:**
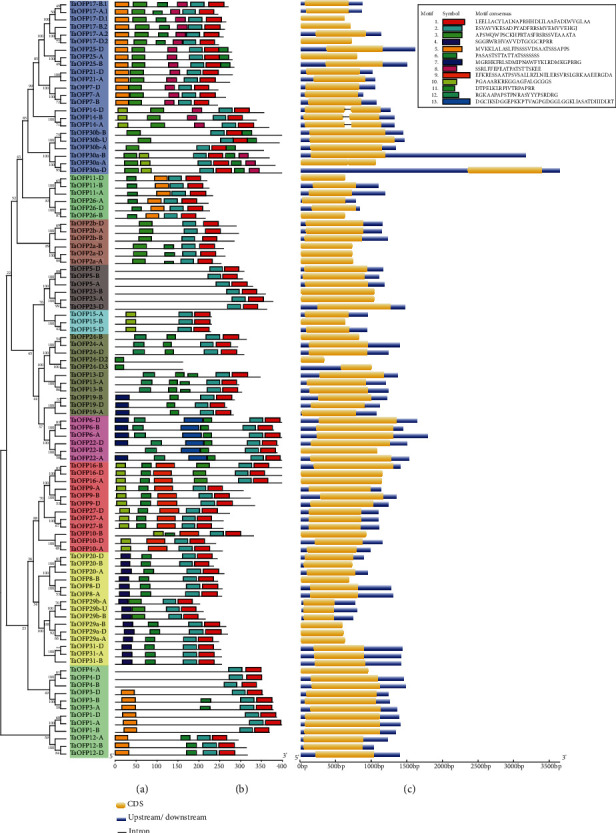
Gene structures and conserved protein motifs of TaOFPs. Phylogenetic relationships, gene structures, and the architecture of conserved protein motifs in *OFP* genes from wheat. (a) Phylogenetic tree constructed based on the full-length sequences of wheat OFP proteins using the MEGA 7.0 software. (b) The motif composition of wheat OFP proteins. The motifs are displayed as different colored boxes. The sequence information for each motif is provided in Supplementary File 4. (c). Exon–intron structures of *TaOFP* genes. Blue boxes indicate untranslated 5′ and 3′ regions, yellow boxes indicate exons, and black lines indicate introns.

To investigate the phylogenetic relationships among rice, Arabidopsis, and wheat *OFP* genes, we constructed a phylogenetic tree based on the sequences of their OVATE domains (Figure 1). The 18 OFP family proteins from Arabidopsis, 31 from rice, and 100 from wheat were classified into four subgroups (I–IV) based on their topological structures (Figure 2). However, most TaOFP and OsOFP proteins were clustered into distinct species-specific clades. Only one group of orthologs was identified among wheat, rice, and Arabidopsis: TaOFP2a, TaOFP2b, OsOFP2, and AtOFP14. These results suggest that the main characteristics of OFP proteins in rice, wheat, and Arabidopsis formed prior to the divergence of monocots and dicots and that they subsequently evolved separately in a species-specific manner.

Members within a specific subgroup exhibited a high degree of amino acid sequence identity (Supplementary File 4). In some subfamilies, *OFP* genes appeared to have expanded in wheat and rice compared to Arabidopsis. For example, subfamily II contains 42 TaOFPs and 14 OsOFPs and only three orthologs (AtOFP7, AtOFP8, AtOFP9) in Arabidopsis. Thus, the *OFP* genes may have evolved to have different functions in monocotyledons and dicotyledons. The various regions of the OFP proteins may be important for their functions.

### 3.3. Genomic Organization and Duplication of Wheat *OFP* Genes

**Figure 3:**
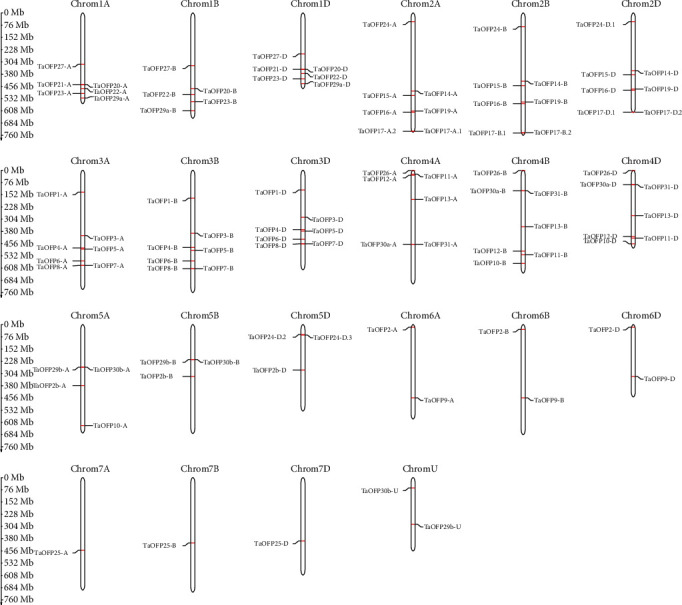
Physical locations of *TaOFP* genes on the *Triticum aestivum* chromosomes. Red dots indicate the positions of *TaOFP* genes.

Physical mapping indicated that the three homologous chromosomes from the wheat A/B/D subgenome contain 100 *OFP* genes (Figure 3), suggesting that the *OFP* genes in wheat were retained on homologous chromosomes during the chromosome doubling process (Supplementary File 5). We detected no clear preference for subgenomes or gene loss. Wheat *OFP* genes are evenly distributed on the three homologous chromosomes (A/B/D) but are unevenly distributed on different chromosomes, with a maximum of seven genes on chromosomes 2, 3, and 4 and only one on chromosome 7A/B/D, regardless of chromosome length (Figure 3). The A homeolog of *TaOFP-10* was translocated from chromosome 5AL to 4AL, which is consistent with previous findings [39, 40]. The A homeologs of *TaOFP12* and *TaOFP30a* were translocated from chromosome 4AS to 4AL and 4AL to 4AS, respectively, which was confirmed by examining the 500-Mb up- and downstream regions of these two genes.

On chromosome 2A/B/D, *TaOFP-17* originated from tandem duplication events. Tandem and segmental duplication are key factors in the generation of new gene family members during evolution. Compared to the genomes of other grasses, inter- and intrachromosomal duplications in wheat are more commonly detected through interspecific whole-genome analysis [41]. Thus, we investigated the segmental and tandem duplication events in the *OFP* gene family in wheat.

We identified three pairs of genes among the 100 *TaOFPs* as tandem duplications and one pair of genes that might have arisen from segmental duplication events, as revealed using Protein Alignment Matrix (Supplementary File 6). Roughly one-to-one correspondences of these tandem duplication and segmental duplication events were observed in the wheat A, B, and D subgenomes; thus, tandem duplication or segmental duplication events often occurred in the same locations in the three wheat subgenomes.

The rate of nonsynonymous (Ka) and synonymous (Ks) substitutions provides a basis for evaluating the positive selection pressure of duplication events, where Ka/Ks = 1 indicates neutral selection, Ka/Ks <1 indicates purifying selection, and Ka/Ks >1 indicates positive selection. We used Ka/Ks Calculator 2.0 to calculate the Ka/Ks ratios of duplicated TaOFPs. The Ka/Ks ratios of three pairs (*TaOFP17-A.1/TaOFP17-A.2*, *TaOFP17-B.1/TaOFP17-B.2*, and *TaOFP17-D.1/TaOFP17-D.2*) of tandemly duplicated genes were 0.287, 0.306, and 0.379, respectively (Table 1), and the Ka/Ks ratio of *TaOFP24-D.2/TaOFP24-D.3*, which were derived from segmental duplication, was 90.65. Therefore, duplication events played a pivotal role in the evolution of *TaOFPs*, and the Ka/Ks average is far less than 1, indicating that *OFP* gene family members have undergone purifying selection.

### 3.4. Analysis of Wheat *OFP* Gene Structures and Conserved Protein Motifs

To study the structures of the *TaOFP* genes, we analyzed their DNA sequences and determined their intron and exon compositions. We used GSDS 2.0 to map the intron–exon structures of the wheat OFP family. Among the 100 *TaOFP* genes, 96 genes (96%) contain no introns, and *TaOFP14-A*, *TaOFP14-B*, *TaOFP14-D*, and *TaOFP30a-A* each contain only one intron (Figure 2(c)). We used MEME to identify the conserved domains of the *TaOFP* gene family members. Gene structure analysis showed that the lengths of the *TaOFP* gene family members were quite similar. The structures of OFP family genes were conserved among various subfamilies, and the number and locations of exons were similar among these genes, suggesting that they have similar functions.

All *TaOFP* genes contain two conserved elements (motif 1 and motif 2) constituting the OVATE domain (Figure 2(b)). Some motifs could be used as markers to identify different subfamilies. For example, motif 8 is only present in subfamily III. These results indicate that during the evolution of the *TaOFP* gene family, internal differentiation might have led to functional differentiation.

### 3.5. Identification of Cis-Elements in the Promoters of *TaOFP* Genes

**Figure 4:**
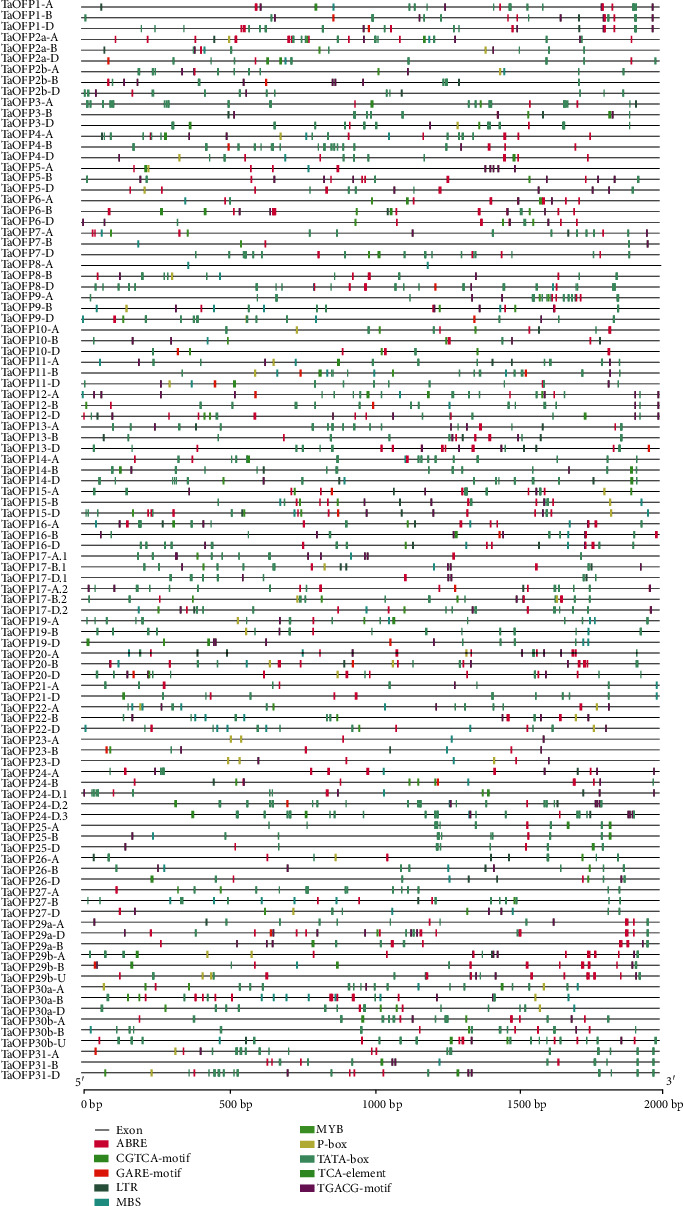
Distribution of major stress-related cis-elements in the promoters of *TaOFP* genes. Putative ABRE, MYB, LTR, MBS, P-box, TATA-box, TCA-element, GARE-motif, TGACG-motif, and CGTCA-motif are represented by different colors as indicated.

We identified putative cis-acting regulatory DNA elements in the promoter sequences of *TaOFPs* genes (2,000 bp upstream of the translation start site) based on the Ensembl Plants database (Supplementary File 7). We analyzed the environmental stress- and hormone-responsive elements further (Figure 4). Almost all of these elements are distributed randomly in the promoter sequences of *TaOFP* genes (Supplementary File 8). Fifty-three genes contain drought-responsive elements (MBS) in their promoters, indicating that the MYB-binding site is involved in drought responsiveness. Forty genes contain temperature-induced response elements known as long terminal repeats (LTRs).

Among the hormone-related cis-acting elements, the MeJA-responsive elements CGTCA and TGACG [42] were the most frequently identified, appearing in 88 *TaOFP* gene promoters. ABA-responsive elements (ABRE) [43] were present in 94 *TaOFP* genes. GA-responsive elements, including the GARE-motif and P-box, were present in 24 and 39 *TaOFP* genes, respectively. These results indicate that the responses of *TaOFP* genes to environmental factors are quite different, suggesting that a complex mechanism controls the expression of these genes in wheat.

However, some cis-elements are distributed in clusters in certain promoters. For example, the MBS-motif, TGACG-motif, motif I, and G-box were detected only in the promoter of *TaOFP4-A*, implying that these elements play essential roles in regulating *TaOFP4-A* expression. The number and distribution patterns of the cis-elements also varied greatly among the promoters of homologous genes assigned the same number, even though their encoded proteins share extremely similar amino acid sequences and domain compositions (Figure 4). For *TaOFP-8*, 15 and 17 TATA-box cis-elements were identified in the promoters of its B and D homologs, respectively, but none were present in the A homolog. These results suggest that the expression of homologs might be regulated by different mechanisms, pointing to their functional divergence during the polyploidization of the wheat genome.

### 3.6. Stage- and Tissue-Specific Expression Patterns of OFP Family Genes in Wheat

**Figure 5:**
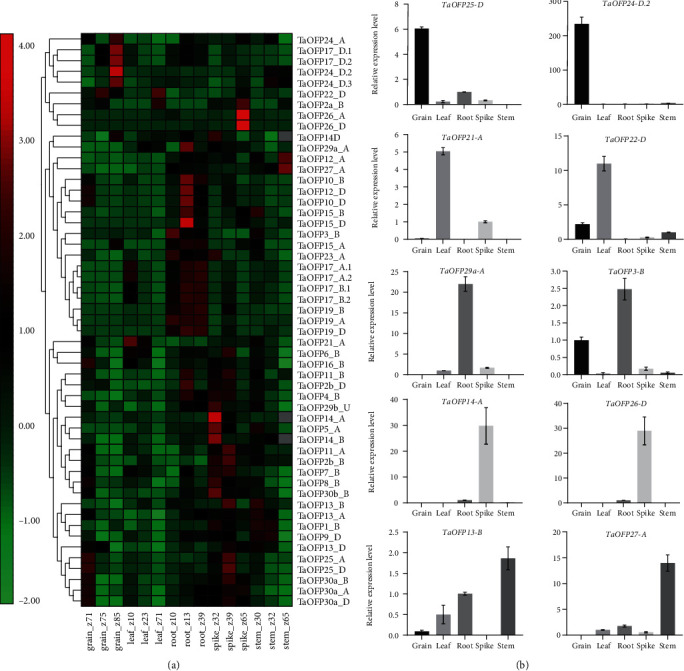
Differential expression of *TaOFP* genes in various tissues and developmental stages. (a) Expression profiles of *TaOFP* genes in various wheat tissues. grain_z71: grains at 2 d after anthesis; grain_z75: grains at 14 d after anthesis; grain_z85: grains at 20 d after anthesis; leaf_z10: leaves at the seedling stage; leaf_z23: leaves at the three-tiller stage; leaf_z71: leaves at 2 d after anthesis; root_z10: roots at the seedling stage; root_z13: roots at the three-leaf stage; root_z39: roots at the flag leaf visible stage; spike_z32: spikes at the second detectable node stage; spike_z39: spikes at the flag leaf visible stage; spike_z65: spikes at anthesis; stem_z30: stems at the heading stage; stem_z32: stems at the second detectable node stage; stem_z65: stems at anthesis. (b) Relative expression levels of *OFP* genes in different wheat tissues under normal conditions. Root: roots at the flag leaf visible stage; stem: stems at the heading stage; leaf: leaves at the seedling stage; spike: spikes at the flag leaf visible stage; grain: grains at 14 d after anthesis. Data represent means ± SD of three biological replicates.

The *TaOFP* genes exhibited various tissue-specific expression patterns, as determined using both wheat transcriptome and qPCR data (Figure 5). Little or no expression of these genes was detected in leaves, whereas they were expressed at high levels in spikes and roots and at moderate levels in grains and stems. *TaOFP1-B*, *TaOFP5-A*, *TaOFP8-B*, *TaOFP14-A/B/D*, *TaOFP16-B*, *TaOFP23-A*, *TaOFP29a-A*, *TaOFP29b-U*, and *TaOFP30b-B* were highly expressed in spikes at the second detectable node stage. *TaOFP2b-B/D*, *TaOFP6-B*, *TaOFP7-B*, *TaOFP11-A/B*, *TaOFP13-B/D*, *TaOFP25-A/D*, and *TaOFP30a-A/B/D* were expressed in spikes at the flag leaf stage.

*TaOFP2a-B* and *TaOFP26-A/D* were strongly expressed in spikes at anthesis. *TaOFP1-B*, *TaOFP3-B*, *TaOFP5-A*, *TaOFP9-D*, *TaOFP13-A/B/D*, *TaOFP15-B*, *TaOFP21-A*, and *TaOFP29b-U* were strongly expressed in stems at the heading stage, and *TaOFP12-A* and *TaOFP27-A* were significantly expressed in stems at anthesis. Conversely, *TaOFP8-B*, *TaOFP10-D*, *TaOFP12-D*, *TaOFP16-B*, and *TaOFP25-A/D* were expressed at the highest levels in grains on the second day after anthesis, whereas *TaOFP17-D.1*, *TaOFP17-D.2*, *TaOFP24-A*, *TaOFP24-D.2*, and *TaOFP24-D.3* were highly expressed in grains at 20 d after anthesis, suggesting that these three genes function in early grain development. *TaOFP2b-D*, *TaOFP4-B*, *TaOFP*10-B/D, *TaOFP11-B*, *TaOFP12-D*, *TaOFP15-A/B/D*, and *TaOFP29a-A* were expressed at high levels in roots at the three-leaf stage. The expression levels of *TaOFP17-A.1/B.1*, *TaOFP17-A.2/B.2*, and *TaOFP22-A/D* in roots gradually increased over time.

To validate the expression patterns of *TaOFP* genes in specific tissues based on the wheat transcriptome data, we examined the expression of *TaOFPs* in five different tissues (root, leaf, stem, spike, and grain) using qPCR. All *TaOFP* expression profiles are shown in Supplementary File 10, and the tissue-specific expression patterns of ten *TaOFPs* are shown in Figure 5(b). *TaOFP24-D.2* was expressed at the highest level in grains at 14 d after anthesis; *TaOFP8-B*, *TaOFP9-A*, *TaOFP14-A*, and *TaOFP26-D* were expressed at the highest levels in spikes at the flag leaf visible stage; *TaOFP25-D* was expressed at the highest level in grains at 14 d after anthesis; and *TaOFP21-A* and *TaOFP22-D* were expressed at the highest levels in leaves at the seedling stage. Furthermore, *TaOFP19-A/B/D*, *TaOFP29a-A*, and *TaOFP3-B* were expressed in roots at the flag leaf visible stage; *TaOFP27-A* was expressed in stems at the heading stage; and *TaOFP13-A/B/D* was mainly expressed in stems. The other *TaOFP* genes were expressed ubiquitously in all tissues or at very low levels. These results indicate that the expression pattern of each *TaOFP* gene is unique and displays strong spatiotemporal and tissue specificity.

### 3.7. Expression Analysis of *TaOFPs* under Stress Conditions

**Figure 6:**
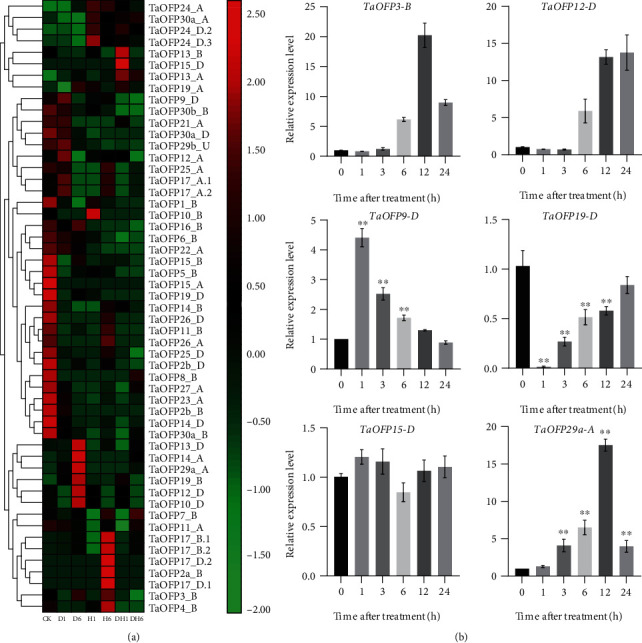
Differential expression of *TaOFP* genes in response to drought and heat stress. (a) Dl and D6: osmotic stress treatment for 1 h and 6 h; H1 and H6: heat stress treatment for 1 h and 6 h; DH1 and DH6: osmotic and heat stress treatments for 1 h and 6 h. (b) Relative expression levels of *OFP* genes under osmotic stress (10% PEG8000). The asterisks indicate statistically significant differences, as determined by Student's *t*-tests (^∗^*P* < 0.05, ^∗∗^*P* < 0.01).

**Figure 7:**
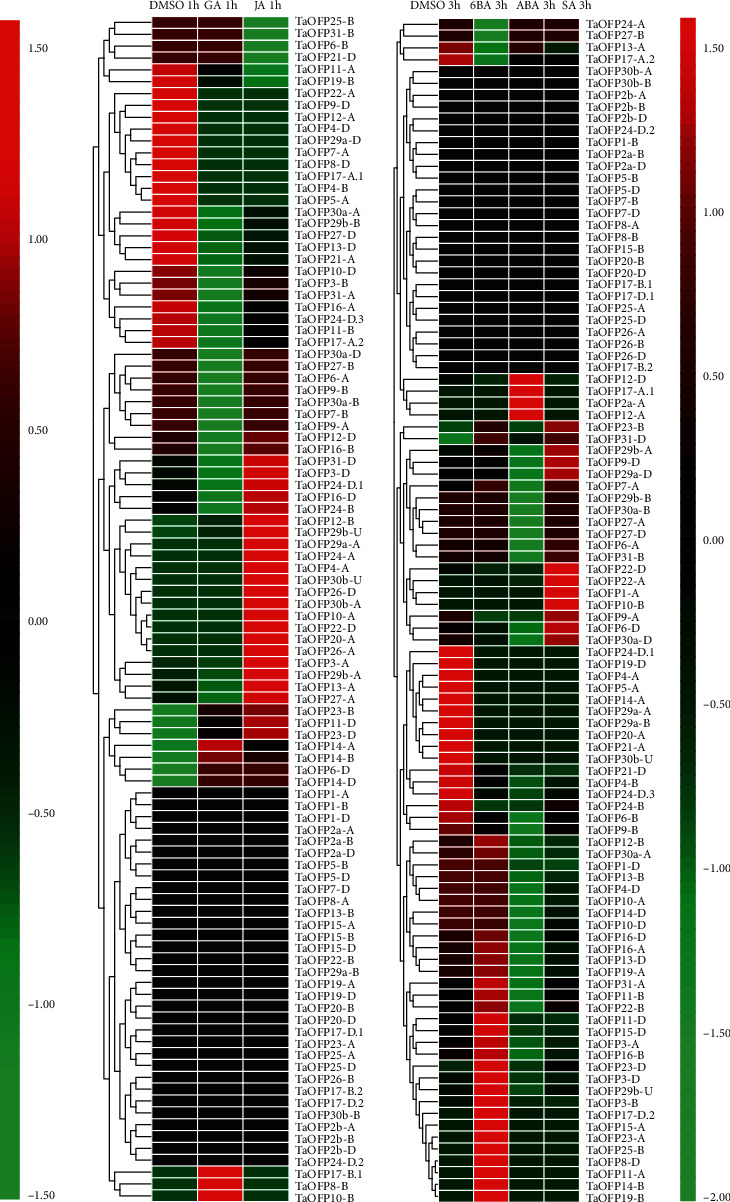
Expression of *TaOFP* gene family members in wheat in response to hormone treatment. DMSO: dimethyl sulfoxide (control); GA: gibberellin; JA: jasmonic acid; 6BA: 6-benzylaminopurine; ABA: abscisic acid; SA: salicylic acid.

To further explore the expression characteristics of wheat TaOFP family genes under suboptimal conditions, we analyzed both wheat transcriptome and qPCR data (Figure 6). Many *TaOFP* genes were responsive to a number of environmental stresses (Figure 7). Different *TaOFP* genes were induced in response to different abiotic stresses such as heat and drought. Here, only genes with more than two-fold differences in transcript levels were considered to be differentially expressed under various treatments. Whereas *TaOFP10-D*, *TaOFP12-D*, *TaOFP13-D*, *TaOFP14-A*, *TaOFP19-B*, and *TaOFP29a-A* were significantly upregulated in response to drought stress, all of the other *OFP* genes were either not expressed or downregulated after drought stress. The expression of *TaOFP2a-B*, *TaOFP17-B.1/D.1*, and *TaOFP17-B.2/D.2* significantly increased in response to heat stress.

To explore the potential functions of *TaOFP* genes that are specifically expressed in roots, we analyzed the effects of drought stress on the expression of these genes by qPCR to investigate their roles in environmental stress responses. Under PEG8000-induced osmotic stress conditions, *TaOFP3-B* and *TaOFP12-D* were upregulated at 6 h of drought stress, and the expression level of *TaOFP3-B* increased to six-fold that of untreated seedlings (Figure 6(b)). *TaOFP29a-A* expression increased during the first three hours of osmotic stress treatment; the greatest increase was detected at 12 h in response to treatment with 10% PEG8000. *TaOFP9-D* expression significantly increased within the first hour of osmotic stress treatment but then declined to control levels by the 24-h time point. *TaOFP3-B*, *TaOFP9-D*, *TaOFP12-D*, and *TaOFP29a-A* were upregulated under osmotic stress and clustered together with *OsOFP8* in clade II, suggesting they might share the same functions; *OsOFP8* was shown to positively regulate drought stress responses [17]. *TaOFP19-D* was downregulated at the beginning of osmotic stress treatment and returned to normal levels after 3 h to 24 h of treatment. We also examined the expression patterns of *TaOFP* genes that are expressed in specific tissues under drought stress, such as *TaOFP15-D*, which is specifically expressed in roots. *TaOFP15-D* was expressed at similar levels under drought stress vs. control treatment (Figure 6(b)). These results suggest that *TaOFP* genes may be involved in regulating a variety of stress responses and are preferentially expressed in specific tissues.

The expression levels of most *TaOFP* genes did not significantly change in response to GA treatment, but *TaOFP6-D*, *8-B, 10-B*, *14-A/B/D*, and *17-B.1* were upregulated by this treatment. *TaOFP1-A*, *TaOFP6-D*, *TaOFP9-A/D*, *TaOFP10-B*, *TaOFP22-A/D*, *TaOFP23-B*, *TaOFP29b-A/B*, *TaOFP29a-D*, *TaOFP30a-D*, and *TaOFP31-D* were significantly upregulated in response to salicylic acid treatment. *TaOFP3-A/D*, *TaOFP4-A*, *TaOFP6-D*, *TaOFP10-A*, *TaOFP11-D*, *TaOFP12-B*, *TaOFP13-A*, *TaOFP14-A/B/D*, *TaOFP17-D*, *TaOFP20-A*, *TaOFP23-B/D*, *TaOFP-24-A/D*, *TaOFP26-A/D*, *TaOFP27-A*, *TaOFP29b-A/U*, *TaOFP29a-A*, *TaOFP30b-A/U*, and *TaOFP31-D* were upregulated in response to jasmonic acid treatment.

*TaOFP3-A/B/D*, *TaOFP8-D*, *TaOFP11-A/B/D*, *TaOFP13-D*, *TaOFP14-B*, *TaOFP15-A/D*, *TaOFP16-A/B/D*, *TaOFP17-D.2*, *TaOFP19-A/B*, *TaOFP22-B*, *TaOFP23-A/D*, *TaOFP25-B*, *TaOFP29b-U*, and *TaOFP31-A* were significantly upregulated following treatment with GA, JA, abscisic acid (ABA), 6BA, or SA. *TaOFP2a-A*, *TaOFP12-A/D*, and *TaOFP17-A.1* were upregulated after 3 h of ABA treatment. Although not all OFP family genes were examined, these results suggest that each OFP family member in wheat has a unique inducible expression profile and thus plays specific roles in plant stress responses.

### 3.8. Overexpressing *TaOFP29a-A* Improves Drought Tolerance in Wheat

**Figure 8:**
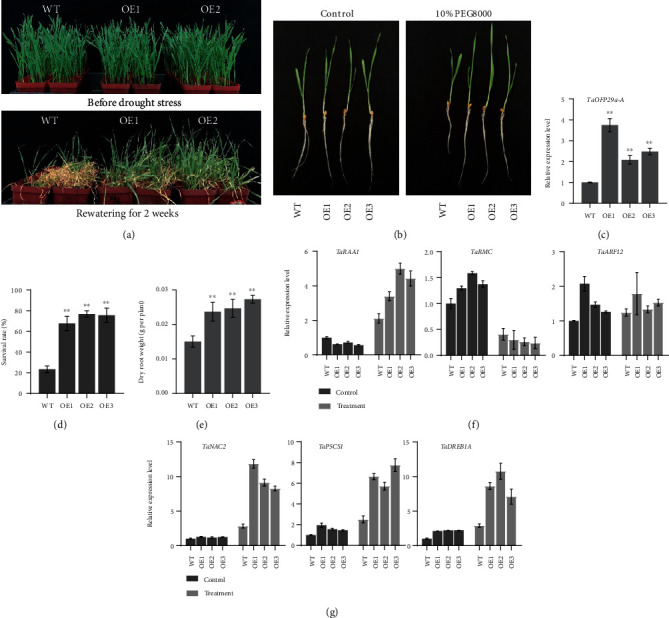
Drought stress responses of transgenic wheat plants overexpressing *TaOFP29a-A*. (a) Phenotypes of transgenic plants before and after osmotic stress treatment. (b) Wheat seeds were germinated in plates containing water for 3 days and transferred to water containing various concentrations of PEG8000 (0 and 10%) for 3 days. (c) The expression levels of *TaOFP29a-A* in transgenic and wild-type plants under normal conditions. (d) Survival rates of drought-stressed wheat seedlings. Each column represents means (±SD) of three independent experiments. (e) Root biomass in 3-week-old plants. Values are means (±SD) of three biological replicates, with each replicate containing 6 seedlings. (f) The expression levels of root-related genes are altered in transgenic plants under drought stress treatment vs. the control. (g) The expression levels of drought-responsive genes are altered in transgenic plants under drought stress treatment vs. the control. Each data point is the mean (±SE) of three experiments (30 seedlings per experiment). Significant differences from the WT are denoted by two asterisks corresponding to *P* < 0.01 by Student's *t*-tests.

We analyzed the role of *TaOFP29a-A* in the drought stress response in wheat based on the results of qPCR analysis (Figure 6). To investigate how increased *TaOFP29a-A* expression enhances drought tolerance in wheat, we generated transgenic lines expressing *TaOFP29a-A* cDNA from wheat cultivar Jingdong18 under the control of the constitutive *ZmUbiquitin1* (*Ubi*) promoter. We analyzed three independent *pUbi:TaOFP29a-A* transgenic lines in the T2 generation. The transgenic lines with enhanced *TaOFP29a-A* gene expression exhibited two- to three-fold higher expression levels of this gene relative to the control (Figure 8(c)). Furthermore, the transgenic plants exhibited higher survival rates than WT plants under drought stress, and their growth recovered to normal levels after rewatering (Figures 8(a) and 8(d)). These results indicate that the transgenic plants had greater drought tolerance than WT plants.

To investigate the potential molecular mechanisms underlying the improved drought tolerance of *TaOFP29a-A*-overexpression lines, we examined 3-day-old transgenic and WT seedlings after 3 days of PEG8000-induced osmotic stress conditions. Compared to WT plants, the transgenic plants had longer roots (Figure 8(b)). The primary root length and dry root biomass of the transgenic plants were markedly greater than those of the WT under water deficit conditions (Figure 8(e)). We examined the expression levels of three auxin transporter genes, *TaARF12*, *TaRAA1*, and *TaRMC*, which are homologs of *OsARF12*, *OsRAA1*, and *OsRMC*, respectively [44–46]; these genes modulate root growth in rice. *TaARF12* was insensitive to drought stress, whereas *TaRAA1* and *TaRMC* were significantly induced by this treatment. Thus, these genes might have contributed to the enhanced development of the root systems of the transgenic plants (Figure 8(f)). These results suggest that the ectopic expression of *TaOFP29a-A* in plants might affect auxin transport and auxin-related plant development.

Finally, we performed qPCR analysis to examine the expression levels of several drought-responsive marker gene in transgenic and WT plants, including *TaP5CS1* [44], *TaNAC2* [47], *TaDREB1A* [48], *TaMYB2A/B/D* [49], *TaDOF* [50], and *TaZAT12* [37, 51]. Under normal conditions, there were no significant differences in the transcript levels of *TaDOF*, *TaMYB2A*, or *TaZAT12* between *TaOFP29a-A*-overexpression and WT plants (data not shown). However, the expression levels of *TaDREB1A*, *TaNAC2*, and *TaP5CS1* were significantly higher in *TaOFP29a-A*-overexpression plants than in the WT (Figure 8(g)).

## 4. Discussion

### 4.1. Wheat OFPs Are Diverse, with a Complex Evolutionary History

Common wheat is a hexaploid species with three closely related subgenomes (A, B, and D). In this study, we identified 100 OFP family genes in wheat encoding 34 TaOFPs, which we named based on sequence analysis and domain composition (File 3). Phylogenetic analysis indicated that homologous genes from different subgenomes encoding a single OFP protein always clustered together, as expected (Figure 1). Not every OFP protein is encoded by three homologous genes in subgenomes A, B, and D. For example, *TaOFP21* includes two homologous genes (*TaOFP21-A* and *TaOFP21-D*), whereas *TaOFP10* lacks homologous genes in the B and D subgenomes (Figure 3). This finding suggests that some homologs might have been lost during long-term evolution and natural selection. Indeed, many studies have shown that in allopolyploid species, genetic variations including gene rearrangements, structural variation, DNA sequence loss or amplification, and transposon activation occurred frequently during genome polyploidization [52]. Alternatively, perhaps there was incomplete coverage in the wheat reference genome.

Gene duplication, fusion, and/or exon shuffling have commonly occurred in eukaryotes, leading to the biological diversity and functional divergence of certain gene families during plant evolution [53–55]. These processes were also involved in the expansion of the *OFP* gene family in plants. In the current study, we identified one *TaOFP* gene pair located within segmental duplication blocks and three pairs (Table 1, Table 2) that were tandemly duplicated.

The typical OFPs share high degrees of sequence similarity in the conserved domains but obvious diversity in terms of gene structure and protein size (file 2, Figure 2). TaOFPs also show very high degrees of sequence similarity in the conserved domains compared to other TaOFPs; although, there are still some differences in the same conserved amino acids (Figure 2), implying that they share a conserved evolutionary relationship. Interestingly, both TaOFP24-D.2 and TaOFP24-D.3 harbor partial OVATE domains (Figure 2). Phylogenetic analysis indicated that TaOFP24-D.2 and TaOFP24-D.3 are closely related to TaOFP24-D.1, suggesting they might have been derived from this typical TaOFP and likely experienced DNA sequence changes during gene duplication. Together, these findings suggest that wheat OFPs have a complicated evolutionary history involving gene expansion and functional divergence.

### 4.2. Functional Prediction of Wheat *OFP* Genes

The *OVATE* gene was first identified as a major QTL controlling pear-shaped fruit in tomato [1, 33]. Subsequently, studies in Arabidopsis and rice indicated that *OFPs* control multiple aspects of plant growth and development [3, 5, 13, 14]. The wheat genome contains more *OFPs* than the Arabidopsis and rice genomes, but the roles of *OFPs* in growth and development remain in wheat remain unclear. *AtOFP1* functions in GA signaling by repressing the expression of *GA20ox1*, a gene encoding a key enzyme in GA biosynthesis, whereas *OsOFP8* is involved in the BR signaling pathway and shows normal responses to GA treatment [17], highlighting the functional diversity of these genes in Arabidopsis and rice. *OsOFP19* modulates plant architecture by integrating cell division patterns and BR signaling [37]. Phylogenetic analysis of *OFP* genes from rice, Arabidopsis, and wheat showed that *TaOFP19* is a homolog of *AtOFP1*. Similar to its ortholog *OsOFP19*, which is expressed in various tissues and in response to GA treatment, *TaOFP19* is specifically expressed in roots and in response to GA treatment. These findings suggest that this gene might share a similar function with *OsOFP19* in the BR or GA signaling pathway.

*OsOFP8* regulates leaf angle via the BR signaling pathway [8]. Overexpression of *OsOFP8* improved drought resistance in rice [17]. The function of *OFP8* is conserved between rice and wheat, as *TaOFP8* positively regulates drought stress responses in wheat. Interestingly, *TaOFP8-B* was downregulated under drought stress (Supplementary File 11), whereas *TaOFP8-A* and *TaOFP8-D* transcripts were not detected; thus, *TaOFP8* plays a different role from its ortholog *OsOFP8* during drought stress. We identified several genes (including *TaOFP8*, *TaOFP20*, *TaOFP31*, *TaOFP29a*, and *TaOFP29b*) of OFP subgroup II that are closely related to *OsOFP8* (Figure 1). Moreover, *TaOFP29a-A* was highly expressed in roots, and *TaOFP29a-A* expression significantly increased under drought stress (Figure 5), whereas *TaOFP29a-B* and *TaOFP29a-D* transcripts were not detected under normal conditions. *TaOFP20*, *TaOFP31*, and *TaOFP29b* were not induced by drought stress treatment (Supplementary File 11). These results suggest that there are some differences between rice and wheat with respect to which *OFP* subfamily genes are induced by a particular stress.

### 4.3. Root-Specific Expression of *TaOFP29a-A* Improves Drought Stress Tolerance in Wheat

We observed that *TaOPF29a-A* was more highly expressed in roots than in other tissues, suggesting that *TaOPF29a-A* might function in root development. In this study, we identified *TaOFP29a-A* and established that it is specifically expressed in roots and upregulated by drought stress. We generated *TaOFP29a-A* overexpression lines to investigate the role of this gene in drought stress tolerance. The *TaOFP29a-A* overexpression lines showed greater tolerance to drought stress than the corresponding nontransgenic plants, including longer roots during germination and greater dry root weight in seedlings, suggesting that the role of *TaOFP29a-A* in regulating root growth might depend on the drought stress pathway.

Optimizing root system architecture (RSA) can overcome yield limitations in crop plants caused by water stress [45]. Many RSA-related genes have recently been identified [56]. In rice, knockout of *OsARF12* led to decreased primary root length [45]. In *OsRMC* RNAi transgenic rice, the primary roots were shorter, and the number of adventitious roots was higher but the number of lateral roots was lower compared to the WT, suggesting that OsRMC might be involved in RSOsPR10-mediated JA signaling [57–59]. Overexpression of *OsRAA1* resulted in reduced primary root growth and an increased number of adventitious roots [46, 60]. To explore the mechanism underlying the role of *TaOPF29a-A* in drought stress, we measured *TaARF12*, *TaRAA1*, and *TaRMC* expression levels in transgenic and WT seedlings under normal conditions or drought stress. The expression patterns of *TaRAA1* and *TaRMC* were consistent with that of *TaOFP29a-A*, whereas of *TaDREB1A*, *TaNAC2*, and *TaP5CS1*, like *TaOFP29a-A*, were upregulated in response to drought stress. These results suggest that TaOFP29a-A might function as a core regulator that activates downstream signaling to maintain drought stress responses at a level that improves plant growth and resistance to water deficit. To date, no downstream or upstream binding proteins of TaOFP29a-A have been identified, suggesting that TaOFP29a-A functions via a complex mechanism that remains to be elucidated. These observations suggest that *TaOFP29a-A* functions in drought stress responses and could potentially be used for genetic engineering to enhance drought tolerance in crops.

## 5. Conclusions

In this study, we comprehensively analyzed the *OFP* gene family in wheat, including their gene structures, evolution, and expression patterns. We also determined that overexpressing *TaOPF29a-A* improved drought tolerance in wheat. These results provide fundamental resources for both evolutionary and functional studies and suggest that *TaOPF29a-A* could be useful for engineering drought-tolerant plants.

## Figures and Tables

**Table 1:** Ka/Ks ratios of tandemly duplicated *TaOFPs.*

Ka/Ks ratios of tandemly duplicated TaOFPs									
Gene name	Phylogenetic cluster	Chr. NO.	Gene name	Phylogenetic cluster	Chr	Ka	Ks	Ka/Ks	Percent identity (%)
*TaOFP17-A.1*	III	2A	*TaOFP17-A.2*	III	2A	0.059244672	0.206248703	0.287249	86.34
*TaOFP17-B.2*	III	2B	*TaOFP17-B.1*	III	2B	0.053410651	0.174226837	0.306558	84.96
*TaOFP17-D.2*	III	2D	*TaOFP17-D.1*	III	2D	0.069567642	0.183393949	0.379334	84.78

**Table 2:** Ka/Ks ratios of segmentally duplicated *TaOFPs.*

Ka/Ks ratios of segmental duplicated TaOFPs									
Gene name	Phylogenetic cluster	Chr. NO.	Gene name	Phylogenetic cluster	Chr	Ka	Ks	Ka/Ks	Percent identity (%)
*TaOFP24-D.2*	I	5D	*TaOFP24-D.3*	I	5D	0.063574285	0.139652008	0.455234	90.65

## Data Availability

All datasets presented in this study are included in the article/Supplementary Material.

## Supplementary Materials

Supplementary 1 Supplementary file 1: Primers used in this study.

Supplementary 2 Supplementary file 2: A complete list of wheat OFP gene sequences identified in the present study.

Supplementary 3 Supplementary file 3: Features of OFP genes identified in Triticum aestivum.

Supplementary 4 Supplementary file 4: Sequence logos for the conserved motifs of wheat OFP domain proteins.

Supplementary 5 Supplementary file 5: Characteristic features of 100 TaOFPs on the chromosome.

Supplementary 6 Supplementary file 6: Percent Identity Matrix of 100 TaOFPs Protein sequence.

Supplementary 7 Supplementary file 7: A complete list of TaOFP promoters sequences identified in the present study.

Supplementary 8 Supplementary file 8: Predicted cis-elements in TaOFP promoters.

Supplementary 9 Supplementary file 9: Transcriptome data of specific TaOFPs wheat tissue specific expression.

Supplementary 10 Supplementary file 10: The expression of TaOFPs in normal 1 and 2.

Supplementary 11 Supplementary file 11: The expression of OFP genes under osmotic stress treatment.
