# Supplementary figures and images for: Genome-Wide Characterization of OFP Family Genes in Wheat (Triticum aestivum L.) Reveals That TaOPF29a-A Promotes Drought Tolerance
Source: Biomed Res Int. 2020 Nov 7;2020:9708324. doi: 10.1155/2020/9708324 (PMC7666709; doi:10.1155/2020/9708324)

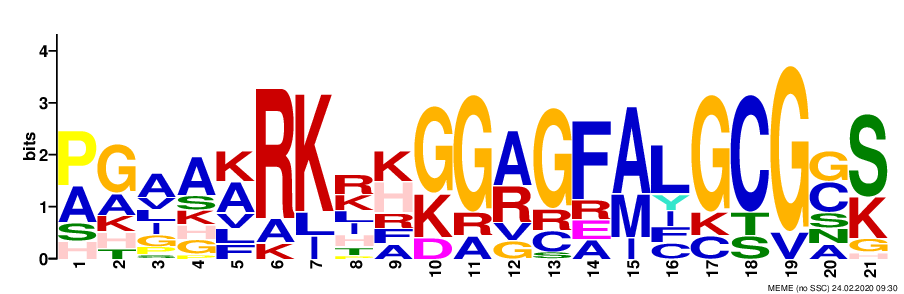

Supplement: Supplementary 4 — Supplementary file 4: Sequence logos for the conserved motifs of wheat OFP domain proteins. [file 9708324.f4.zip › Supplementary file 3-Sequence logos for the conserved motifs of wheat OFP domain proteins/10logo.png]

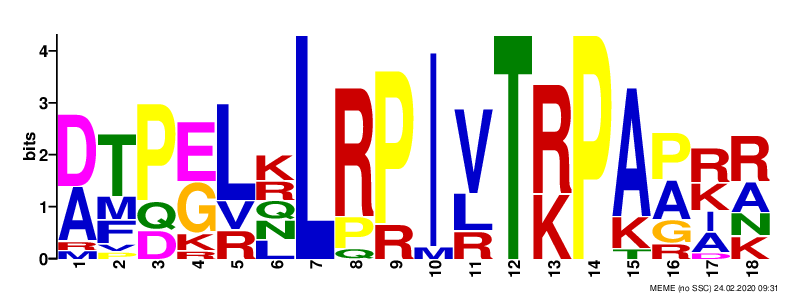

Supplement: Supplementary 4 — Supplementary file 4: Sequence logos for the conserved motifs of wheat OFP domain proteins. [file 9708324.f4.zip › Supplementary file 3-Sequence logos for the conserved motifs of wheat OFP domain proteins/11logo.png]

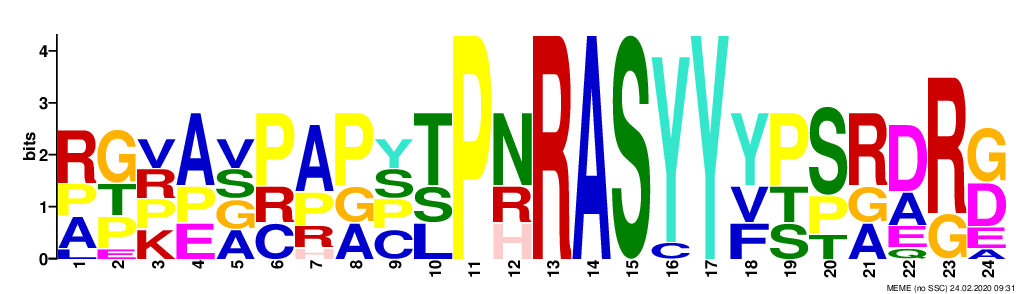

Supplement: Supplementary 4 — Supplementary file 4: Sequence logos for the conserved motifs of wheat OFP domain proteins. [file 9708324.f4.zip › Supplementary file 3-Sequence logos for the conserved motifs of wheat OFP domain proteins/12logo.png]

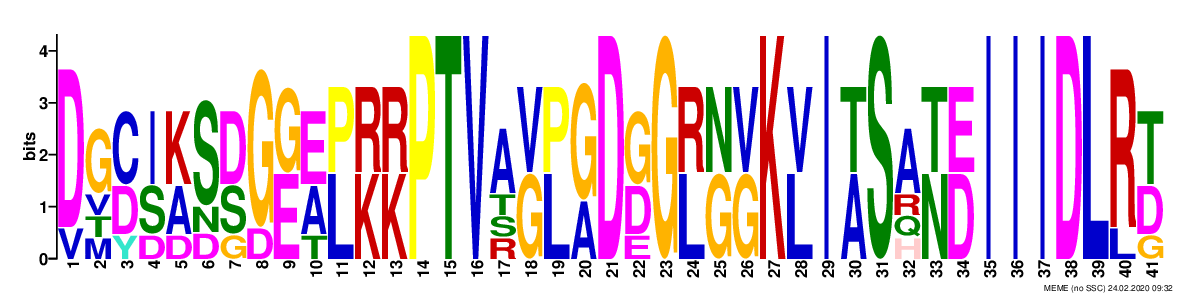

Supplement: Supplementary 4 — Supplementary file 4: Sequence logos for the conserved motifs of wheat OFP domain proteins. [file 9708324.f4.zip › Supplementary file 3-Sequence logos for the conserved motifs of wheat OFP domain proteins/13logo.png]

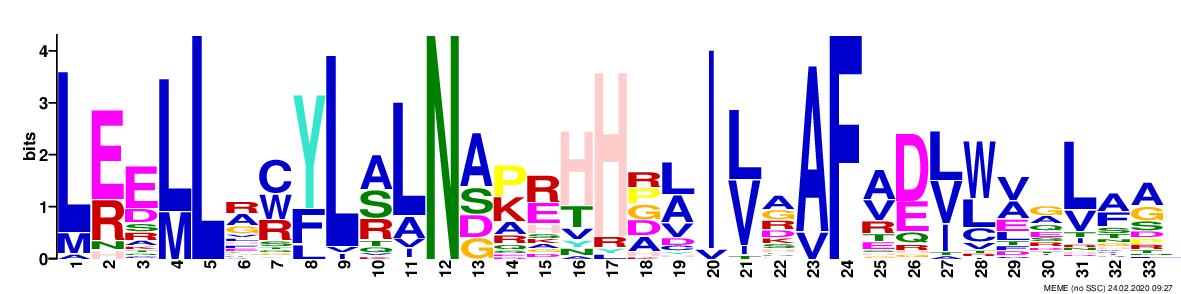

Supplement: Supplementary 4 — Supplementary file 4: Sequence logos for the conserved motifs of wheat OFP domain proteins. [file 9708324.f4.zip › Supplementary file 3-Sequence logos for the conserved motifs of wheat OFP domain proteins/1logo.png]

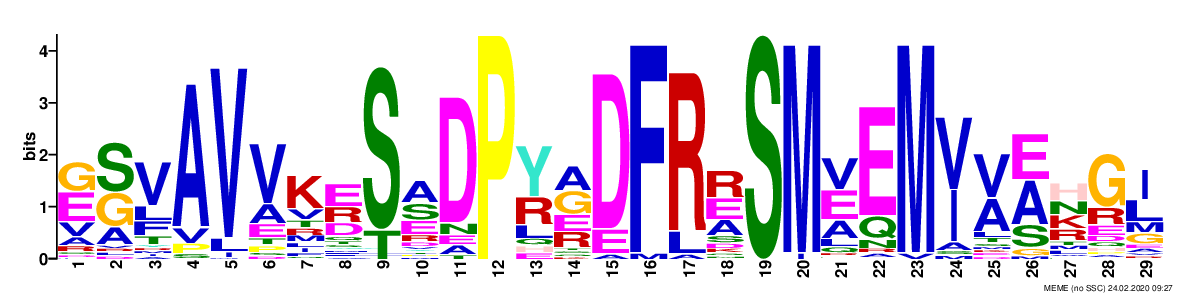

Supplement: Supplementary 4 — Supplementary file 4: Sequence logos for the conserved motifs of wheat OFP domain proteins. [file 9708324.f4.zip › Supplementary file 3-Sequence logos for the conserved motifs of wheat OFP domain proteins/2logo.png]

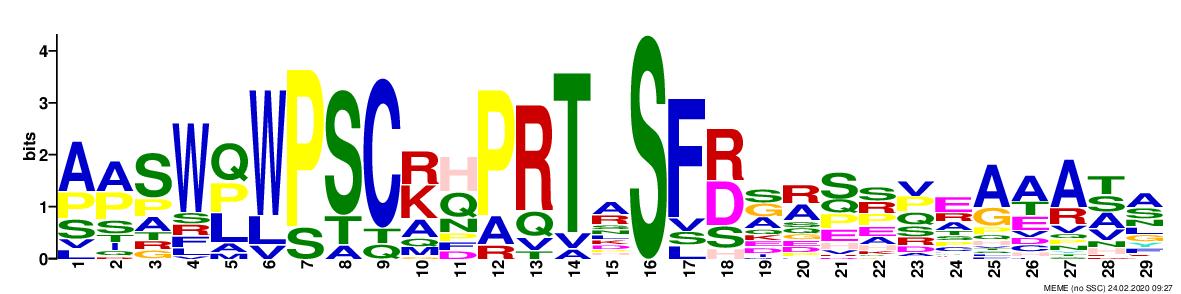

Supplement: Supplementary 4 — Supplementary file 4: Sequence logos for the conserved motifs of wheat OFP domain proteins. [file 9708324.f4.zip › Supplementary file 3-Sequence logos for the conserved motifs of wheat OFP domain proteins/3logo.png]

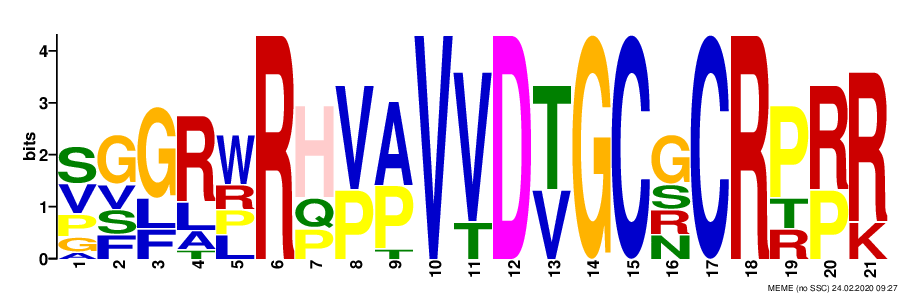

Supplement: Supplementary 4 — Supplementary file 4: Sequence logos for the conserved motifs of wheat OFP domain proteins. [file 9708324.f4.zip › Supplementary file 3-Sequence logos for the conserved motifs of wheat OFP domain proteins/4logo.png]

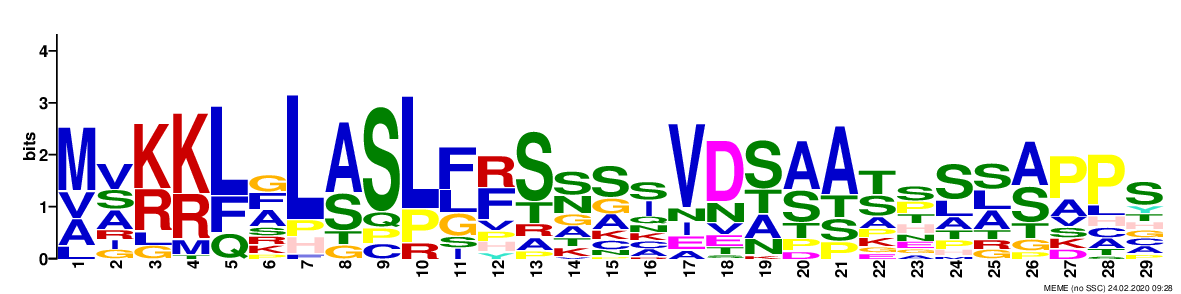

Supplement: Supplementary 4 — Supplementary file 4: Sequence logos for the conserved motifs of wheat OFP domain proteins. [file 9708324.f4.zip › Supplementary file 3-Sequence logos for the conserved motifs of wheat OFP domain proteins/5logo.png]

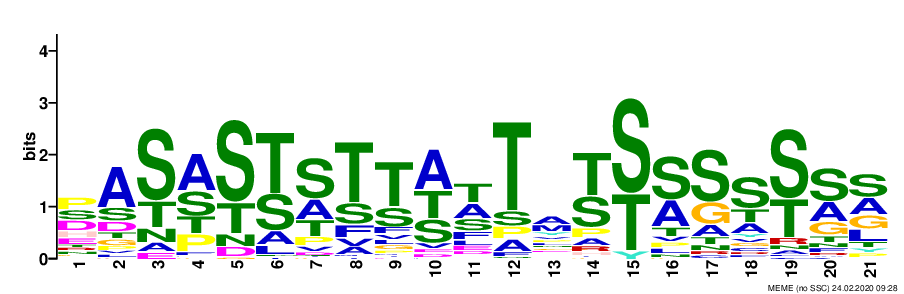

Supplement: Supplementary 4 — Supplementary file 4: Sequence logos for the conserved motifs of wheat OFP domain proteins. [file 9708324.f4.zip › Supplementary file 3-Sequence logos for the conserved motifs of wheat OFP domain proteins/6logo.png]

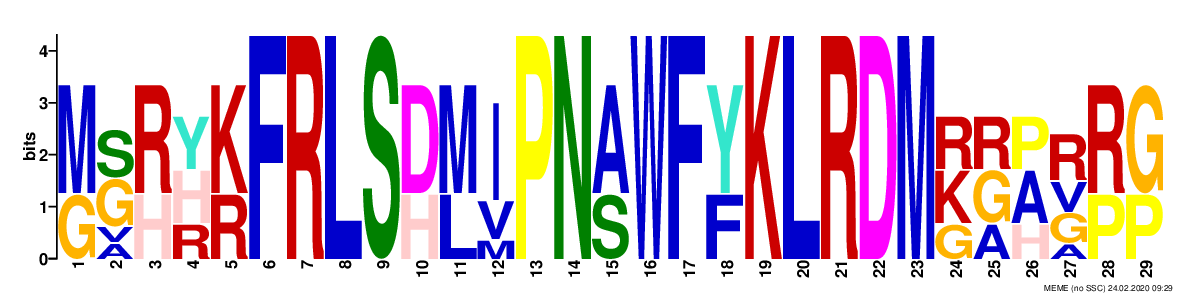

Supplement: Supplementary 4 — Supplementary file 4: Sequence logos for the conserved motifs of wheat OFP domain proteins. [file 9708324.f4.zip › Supplementary file 3-Sequence logos for the conserved motifs of wheat OFP domain proteins/7logo.png]

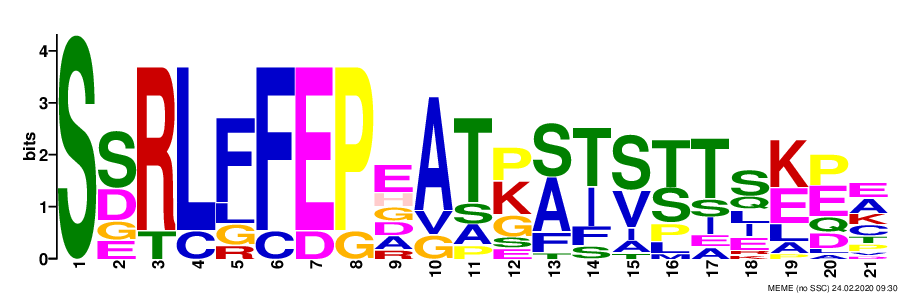

Supplement: Supplementary 4 — Supplementary file 4: Sequence logos for the conserved motifs of wheat OFP domain proteins. [file 9708324.f4.zip › Supplementary file 3-Sequence logos for the conserved motifs of wheat OFP domain proteins/8logo.png]

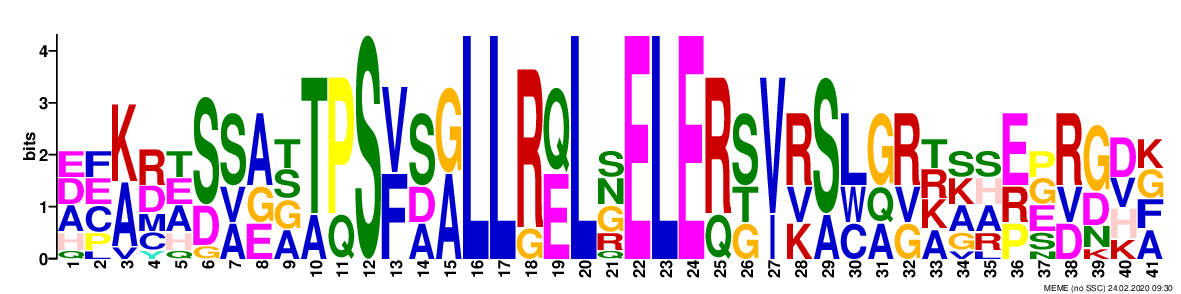

Supplement: Supplementary 4 — Supplementary file 4: Sequence logos for the conserved motifs of wheat OFP domain proteins. [file 9708324.f4.zip › Supplementary file 3-Sequence logos for the conserved motifs of wheat OFP domain proteins/9logo.png]
